# The New Food

**Published:** 1870-12

**Authors:** 


### THE NEW FOOD,

Cheap, nutritious and healthful, beyond almost any other article which comes upon our tables, merits universal attention. It is a Farina of the nature of Arrowroot, Sago, Tapioca, Maizena, Corn Starch, and other foods of that character, but superior to any of them in

### CHEAPNESS, because it not only costs less than one half of any other farina, but it is largely more nutritious, for one pound of it contains very nearly twice as much material for the support of the system as roast beef.

### HEALTHFULNESS.

Without being medicinal, it contributes more towards the maintenance and restoration of the health than any drug can possibly do, because by its light, mild, mucilaginous nature, it rests on the stomach, and is retained to impart nourishment and strength promptly, when even the mildest liquids are rejected.

It is peculiarly appropriate in those cases of weak stomach and irritable bowels of teething children, for it soothes while it sustains, removing the inflammatory condition, bringing about a healthful action, giving strength and vigor before ordinary foods are half digested. For the same reason it is applicable to a variety of cases of

### DYSPEPTIC DISEASE

where the stomach is so weak, that although the patient has a voracious appetite, he is conscious that his food does him little or no good, does not give him strength; this is because the stomach has lost its power to draw nutriment from the food taken; but the new food is so wholly nutritious, and such a small amount of it imparts strength, that the patient begins to "gain" right away; because from its light and mild nature, it is so easily prepared to impart its sustaining qualities to the system. The New Food, having these valuable qualities, is prepared from Irish or Iceland Sea Moss, which for more than a century has been prescribed by educated physicians in all countries for its supposed medicinal virtues, in coughs, colds, chest affections, and in all scrofulous diseases, the benefits derived from its use being in reality owing to the readiness with which its nutrient particles are prepared for giving warmth, strength, and flesh to the system.

Rand's Sea Moss Farine keeps good for years in a dry place, and is used in such a variety of dishes, that there is not a household in the land which will not find it a very great convenience to keep a stock of it on hand all the time, especially as it can be prepared for the table in a very few minutes, in a great variety of forms, in the shape of

| | |
|---|---|
| Blanc Mange, | Jellies, |
| Charlotte Russe, | Puddings, |
| Custards, | Pies, |
| Creams, | Soups, |
| Gruels, | Starches, |

Apple Farina, &c.

N. B.—Since the above was put in print, a letter has been shown us from C. S. Sheldon, M.D., of New Jersey, in gratifying confirmation of the efficacy of Rand's Sea Moss Farine in the teething and summer complaint of infancy. From the excessive and wasting diarrhœa, and an irritability of stomach which would not allow the blandest food known to remain a moment, the little sufferer was wasted to a skeleton, and given over to die, when the physician bethought himself of trying a small quantity of Rand's Sea Moss Farine, mixed with a little water—it was retained—a little more was given; from that hour the child began to rally without medicine, and was saved.

